# Analyzing the Components of Emotional Competence of Football Coaches: A Qualitative Study from the Coaches’ Perspective

**DOI:** 10.3390/sports6040123

**Published:** 2018-10-23

**Authors:** Honggyu Lee, Hagen Wäsche, Darko Jekauc

**Affiliations:** 1 Institute of Sport Science, Goethe University Frankfurt am Main, 60487 Frankfurt am Main, Germany; 2 Institute of Sports and Sports Science, Karlsruhe Institute of Technology, 76131 Karlsruhe, Germany; hagen.waesche@kit.edu

**Keywords:** emotional competence, circular emotional process, football coach

## Abstract

Emotional Competence (EC) is regarded as a fundamental skill for sports coaches. However, the applications of EC in football coaching are not well understood. This study analyzed the specific emotional processes football coaches experience. We interviewed 18 football coaches and analyzed the interview transcripts by using a systematic analysis process based on Grounded Theory principles. We derived a model from this analysis that comprises a four-phase process: emotional triggers, emotional experiences, emotion regulation strategies, and emotional consequences. In this model, we identified four categories which act as triggers of emotions in football coaches. These emotions can be positive or negative and are manifested at three levels. However, the coaches vary in their capability to perceive emotions. Our model also shows that coaches’ emotion regulation strategies influence the effect of emotional experiences. Experienced emotions promote consequences with psychological and social implications for coaches and may influence their perception of future situations. In short, the process seems to be circular. This finding suggests that the ability to deal with emotions is an important aspect for football coaches.

## 1. Introduction

The ability to deal with emotions is a fundamental skill for coaches, since emotions have wide-ranging effects in sports. They impact psychological well-being, social relationships, and performance [1]. It is particularly important for football coaches to embrace the responsibility of caring for their own emotions and the emotions of their players [2]. For example, when football coaches receive continuous feedback, such as praise or criticism concerning themselves as a person or their teams’ performance, they may experience corresponding positive and negative emotions. Coaches who cannot control their own emotions have a higher chance of suffering from physiological and psychological problems [3]. This raises an important question: How do emotional processes work in the context of football?

Oately and Jenkins [4] mentioned this affect is a psychological term which embraces emotion, feeling, mood, and personality traits. Emotion can be understood as the determinant of feeling, mood, and personality traits. Scholars have explored emotions from various angles over the past few decades and have studied emotional triggers [5], types of emotions [4], regulation of emotions [6], the effects of emotions [7], and emotional processes [8]. Emotional functionality (i.e., which emotion is effective for sports performance) is particularly relevant in sports, since positive and negative emotions are related to sports performance [9]. For example, when a coach or a player maintains the optimal emotional state, their performance increases. On the other hand, when they are in an adverse emotional state, their performance decreases. Therefore, the ability to handle emotions is an important psychological ability for coaches [10].

This ability is called Emotional Intelligence (EI); however, recently some researchers used Emotional Competence (EC) as the preferred label instead of EI [11,12]. EC is defined as the ability to monitor, identify, and utilize one’s own emotions and the emotions of others [13]. Scholars considered EC from two perspectives: ability (i.e., what people can do) and traits (i.e., what people do) [14]. Mayer and Salovey [15] conceptualized EC with respect to ability, focusing only on mental skills and dividing them into four categories: recognition of emotion, facilitation of emotion, understanding of emotion, and regulation of emotion. Meanwhile, by Petrides’ [16] definition, the trait perspective views basic emotional processing capacity as critically dependent on individual personality traits; each person has individual tendencies to behave in particular ways. Petrides’ model has four dimensions: emotionality, self-control, sociality, and well-being. Another study proposed a three-level model that attempts to determine the relationship between the ability and trait models. In particular, it asserts that people’s emotional processing depends on their emotional knowledge, ability, and traits [14].

George [17] promoted EC as fundamental for success in leadership, asserting that the literature on leadership had given little attention to the emotions of leaders. Scholars have also examined the evidence of EC’s importance in various areas, including pedagogy [18], nursing [19], business [20], and sport [21]. These studies pointed out that EC is related to success and well-being. Wong and Law [20], for example, discovered that people with higher EC are more successful in their jobs and have higher levels of well-being than the people with lower EC.

Meyer and Fletcher [22] have proposed that EC is related to the satisfaction of the coach and the player, performance improvement, good relationships, and effective leaderships. Scholars have published several studies on EC in the context of sports. These studies have focused on the relationship between the players’ EC and their performance [23], the relationship between the players’ EC and their emotional regulation [24], the promotion of EC programs for players [25], the development of EC questionnaires [26], EC and sports organization [27], and EC and coaching [28,29].

A recent review of research on EC in sports science [21] revealed that most studies have examined the EC of players involved in different types of sports (e.g., team and individual sports), focusing on the relationship between players’ EC and other factors (e.g., performance and emotional regulation). Although scholars regard EC as a fundamental factor for successful coaching [30], the topic of coaches’ EC has received little attention.

Several studies on coaches’ EC have examined the possibility of applying EC in coaching [22,30]. Researchers have examined which model (e.g., ability or trait) is better suited in the field of coaching. Since these studies did not reflect the perspectives of practicing coaches, researchers have highlighted the need for a qualitative research on coaches’ EC that considers their perspectives [22]. Wagstaff et al. [27] undertook a qualitative study on the components of EC in the domain of sports organization. They determined identification of emotions, comprehensive knowledge of emotions, and management of emotions as the components of EC. They acknowledged that their findings did not reflect on the participants’ specific professions and sporting codes, despite the confirmed importance of differentiating between different types of sports [31].

The practical application of research-generated knowledge has been an important goal of sports psychology [32]. To achieve this goal, researchers must consider the type of sport as a significant factor because the appropriate emotional state for optimal performance varies from sport to sport [9]. For example, previous studies examined the optimal emotional state for performance among the players in various types of sport [33]. Campo et al. [34] noted that inconsistent findings stem from the varied natures of each sport. A previous review study that focused on football [31] also reflected in this perspective. Thus, to improve practical transferability, scholars should be aware of the specific nature of the sport under examination. No previous studies have considered the EC of football coaches; as a result, little is known about how football coaches process emotions and how emotions affect their work.

This study explores typical situations in which emotional experiences of football coaches occur, how they deal with their own emotions and what type of consequences emerge for their work. We aim to generate utilizable knowledge regarding intrapersonal EC in football and sports science for practicing coaches. Moreover, we hope that our findings will contribute to the development of a program that focuses on football coaches’ EC.

## 2. Method

### 2.1. Participants

Football coaches were contacted by email invitations to participate in the study. Students who were themselves football coaches referred to using the website listing of football club coaches. Furthermore, these coaches referred additional colleagues to participate. We selected 18 male football coaches through this process. These coaches work as head coaches in German semiprofessional and amateur football leagues for both youth (identification number range from I9 to I18) and adult teams (identification number range from I1 to I8), and were all licensed by the German Football Association. The selected football coaches were: their age range of 20–58 years old with the average age *M*_age_ of 40.56 years old, and their experience range of 4–40 years with the average coaching experience *M*_coaching experience_ of 17.17 years.

### 2.2. Data Collection

This study was approved from the Ethics Committee of Goethe University Frankfurt am Main. We conducted semi-structured interviews based on an interview guide [35], in order to understand and collect information about the practices of football coaches. The initial interview questions are based on our understanding and knowledge from previous studies of emotional processes and EC (e.g., trigger, perception, regulation and outcome of emotion) [8,10,13]. The interview guide was developed by the researchers and was pilot-tested for the clarity of the questions. At the start of each interview, we informed participants that we would record the session, explained the study’s general format and procedure, and assured the participants that we would use the data anonymously. We conducted the interviews by asking open-ended questions. The first part of the guide’s questions related to individual’s interests (e.g., Who is your favorite coach?) to build rapport. In the second part, we asked participants to recall situations which they had experienced as football coaches that triggered positive and negative emotions. The coaches were explicitly invited to freely describe their experiences in these situations. During the interviews, we used “how”—questions based on the information given by the coaches. These questions included the following: How did you recognize your emotions? How did you regulate your emotions? How did these emotions affect you? How would you regulate such emotions if they recurred? These questions were asked to gather detailed information that can be helpful for understanding the individual perspective [35]. All interviews lasted between 20 min and 45 min and were transcribed.

### 2.3. Data Analysis

**Figure 1:**
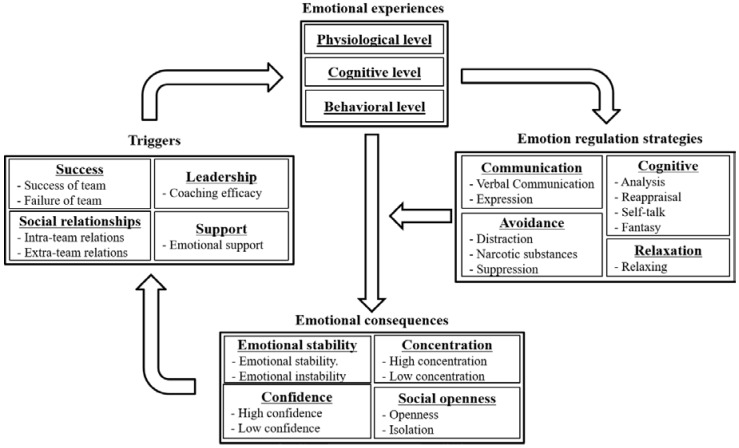
The cyclic model of emotional processes of football coaches.

Studies have suggested that researchers should examine specific components of EC in sports in order to effectively understand and apply EC in the field of sports [22,36]. We analyzed transcripts of the interviews using a systematic analysis method based on Grounded Theory principles, which consists of inductive and deductive analyses, and which is useful for discovering and understanding psychological and social phenomena in natural contexts [37]. This method involved a three-stage process. The first stage was open coding; we used line-by-line coding to conceptualize the raw text data of the interviews and then compared that data to other concepts in order to categorize relevant information with similar meanings. The raw text data were assessed by the researchers to identify the gaps between the concepts. The second stage involved axial coding, which we used to check the connections between each concept we established in the coding paradigm. In this stage, we grouped the identified concepts into sub-categories (e.g., success, social relationships, leadership, and support) within the component triggers (Figure 1). The final stage involved selective coding to determine core categories and to integrate each category (e.g., triggers, emotional experiences, emotion regulation strategies and emotional consequences). We iteratively followed these three coding procedures, not only from open coding to selective coding, but also from selective to open coding, until we determined we would not collect any fresh knowledge. After the presentation of the model, we discussed its components and underlying processes in detail. To eliminate preconceptions and other potential biases, we conducted weekly meetings to compare and discuss the distinctions between the categories. To enhance the credibility of our study, we invited another psychologist to check our categories.

## 3. Results

Based on the data collected from the practical experiences of 18 coaches, a final model was developed that comprises an overview of the main findings (see Figure 1). As a main result of the qualitative analysis, we identified four components of emotional processing, which relate to each other and form a four-phase process: triggers, emotional experiences, emotion regulation strategies, and emotional consequences. The analysis revealed that the football coaches face four triggers: success, social relationships, leadership, and support. These triggers make the coaches experience emotions, which can be positive and negative and which manifest at the physiological, cognitive, and behavioral levels. However, the capability to perceive emotions differs from coach to coach (emotional experiences). Coaches apply various emotion regulation strategies which they know and typically use, that is, communication, avoidance, cognitive and relaxation strategies, which influence their experiences of emotions (emotion regulation strategies). Finally, we found that regulated emotions moderate emotional consequences. In our interviews, we identified emotional stability, confidence, social openness and concentration as emotional consequences. In turn, these emotional consequences may influence the coaches’ perceptions of situations that may trigger emotional states. In short, the emotional processing appeared to be circular.

### 3.1. Triggers

We identified four main categories as triggers of coaches’ emotional experience: success, social relationships, leadership, and support.

#### 3.1.1. Success

All coaches, who participated in the study, described issues concerning the first trigger category, which is success. Success represents a coach’s appraisal of the team’s performance during a season. Success is related to expectations which can neither be met nor be exceeded. Additionally, the coaches gauge success based on whether the player and team performances are improving or not. This category has two opposite poles: team success and team failure.

Ten coaches expressed that team results immediately impact their emotions. For example, winning matches triggers positive emotions (e.g., joy and happiness). One coach described the emotions associated with winning matches as follows:

I have celebrated four promotions with my teams and every single promotion was very happy for me.

(I4)

Eleven coaches mentioned that recognizing improved team performance triggers positive emotions. One coach expressed his emotional reaction to the development of his team as such:

The positive situation is very easy. When a team gets along well and works together well, it triggers positive feelings in me. When I see that the players really develop well after half a year or after one year, it makes me feel positive. I know there are many coaches who have a tendency towards result-oriented football. For those coaches, the most important thing is simply victory. This is not the case for me. Victories do not give me any positive feelings.

(I9)

The above comments suggest that quality of performance can have a significant impact on this coach’s emotions. In other words, football coaches value both the results (i.e., what the result of a game is) and quality of performance (i.e., how their teams are playing). The following quotation from another coach underlines this finding:

We had to play against an overpowering team. The players on the field from the other team were much strong than ours. Some of our players are technically really bad. But my players tried their best with wisdom and character. At least, we could defend ourselves and leave the field with dignity. We lost 4:0; however, I was happy, and so far, it was really my favorite game with this team.

(I6)

In a similar vein, eight coaches experienced negative emotions (e.g., sadness) when they suffered failures, including poor team performance or defeat. One coach commented on his team’s poor performance:

I feel bad when I see that my team does not implement the content of the training, even though we had frequently talked it through.

(I12)

Another coach described losing a game as follows:

We were tenth in the league table and lost 0:5 against the last team in the league table. I was very depressed and disappointed after this game.

(I11)

#### 3.1.2. Social Relationships

Ten out of the 18 coaches in the study referenced social relationships as the second trigger. This category relates to a quality of a coach’s individual relationships with players. It also encompasses the attitudes of other participants in football, including players and coaches from other teams. The category thus has two aspects: intra-team relations and extra-team relations.

Five coaches frequently mentioned that the quality of relationships with players as an emotional trigger. These coaches noted that close relationships with their players trigger positive emotional experiences (e.g., joy and happiness). One coach described the close relationships with his players as follows:

It triggers great happiness in me, when my players come to training with a smile, or when they tell me something relating to small things from school.

(I15)

On the other hand, coaches experienced negative emotions (e.g., sadness) when players show negative attitudes toward them. One coach explained his experience with inattentive players as follows:

When I, as a coach, have the feeling that the team hasn’t listened to me, I’m a bit disappointed.

(I5)

Eight coaches also took their relationships with other people involved in football into an account. They specifically reacted negatively to situations that they considered unethical, unfair, or disrespectful words and behavior contradicting their conception of football culture. In other words, they regarded sportsmanship as an important component in football. For example, being insulted or abused by the coaches and players of other teams or having to respond to racism directed towards their teams induced negative emotions (e.g., fear and anger). One coach highlighted the difficulty of seeing his players being treated disrespectfully:

It was the worst for me when the players from the opposing team seriously insulted our players with foreign backgrounds and were racist to them. This does not belong on the football field.

(I7)

#### 3.1.3. Leadership

Five of the eighteen coaches in this study mentioned issues referring to the third category, leadership. This trigger refers to coaches’ intentional and goal-oriented ability or inability to directly influence the performance of their teams in pursuit of common goals. This category has one aspect: coaching efficacy.

The coaches felt responsible for providing the support and leadership necessary to enable their players to perform well on the field. Three coaches said that recognizing the efficacy of their advice or guidance in player and team performance triggered positive emotions (e.g., joy and happiness). One coach described the experience of effective coaching in this way:

Yes, it is a great feeling because I recognized that, with some methods, I can properly affect my players.

(I3)

Correspondingly, two coaches noted that perceiving lack of their experience or ideas for effectively managing their teams produced negative emotions (e.g., fear and sadness). One coach made the following remarks about how his lack of skill in managing his team affected his emotions during the game:

The trigger of disappointment was, of course, the poor performance of my team. I could do nothing while my team was performing poorly, and I could not even guide my team to win the game.

(I14)

#### 3.1.4. Support

One of the eighteen coaches referenced the relationship between support and negative emotions. We defined this trigger as the struggle of the relevant people in their work environment. Football coaches, who receive negative feedback from the players’ parents and inadequate emotional support from the club manager, feel negative emotions (e.g., helplessness). The following quotation encapsulates his experience of insufficient support:

I feel a certain sense of helplessness. When my coaching style was criticized by the parents, the football club supported me. However, this only helped me internally and not externally. Thus, I stood between the club and the parents.

(I10)

### 3.2. Emotional Experiences

The four trigger categories observed in the study impacted the emotional experiences of coaches. Emotional experience represents the coaches’ perceptions of their emotional responses to different situations in football. Not only could we find out that eight of the 18 football coaches interviewed experienced positive (e.g., joy and happiness) and negative emotions (e.g., anger, sadness, helplessness, and fear), but also that their emotions manifested themselves physiologically, mentally, and behaviorally. Therefore, we quantified emotional experience in terms of physiological, cognitive, and behavioral levels.

#### 3.2.1. Physiological Level

Four coaches reported experiencing physiological responses to the triggers. These responses helped them to accurately perceive their emotions. The physiological changes include: goose bumps, sweating, tremors, increased blood pressure, and increased pulse rates. The following quotation exemplifies one coach’s description of the physiological changes:

Of course, I recognize the anger physically. Just by the pulse rate going up, I know that I’m angry.

(I9)

Another coach pointed out that the physiological change helped him understand the emotion he was experiencing:

I have noticed that I feel the increased blood flow in my body and that it just goes up into my head. It makes me recognize my emotions quickly. There is a certain pressure in my head. This is actually the feature by which I recognize my emotion.

(I17)

#### 3.2.2. Cognitive Level

Four coaches reported experiencing cognitive changes in response to the emotion-triggers. These coaches described how an emotionally triggering situations lingered in their thoughts. In other words, they were fixated on the triggers of the emotional experiences. The following quote illustrates this type of cognitive change:

I think about the fact that I’m actually a teacher in high school, I have a family, but I devote 88% of my thoughts to the football players.

(I6)

Another coach described his cognitive state in this way:

I think that the lost game is reflected again and again in my environment. I have thought about many things after the game…. I might think about it for 1–2 days.

(I11)

#### 3.2.3. Behavioral Level

Four coaches reported experiencing behavioral responses to emotional experiences. Behavioral responses encompass expressive external, and nonverbal actions, such as changes in facial expression, posture, and physical action. The coaches described experiencing positive and negative situations that elicited smiles, tears, changes in the tone of voice, and active action. Explaining the relationship between positive emotions and active actions, one coach commented:

I feel glad and I start to cheer or…. Perhaps I move five meters forward to release the feeling of success. I think that these are the emotions.

(I8)

Another coach described how his facial expression responded to positive emotions:

I was happy about it. I expressed my happiness by laughing, with my words, my facial expression, and my gestures.

(I14)

In total, eight football coaches reported that their various emotional responses helped them to understand the emotions they were experiencing. However, 10 coaches did not share this sentiment. They had a more limited comprehension of their emotional experiences. For instance, some coaches claimed to find it difficult to recognize and describe their emotional experiences. One coach remarked:

I don’t know what happens in the brain or what happens in the body.” (I1). Another coach expressed a similar view: “I am not that emotional. Therefore, it is hard to identify emotion…

(I18)

The following quote further illustrates the difficulty of describing one’s emotion:

I cannot find the words to say how I feel. So, in my opinion, this emotion cannot be expressed in words.

(I4)

The above perspectives suggest that abilities to recognize and identify emotional experiences can vary from one football coach to another. In addition, some coaches mentioned that the emotions they experienced promoted emotional consequences. This supports the notion that the recognition of emotional experiences directly relates to emotional consequences. This point will be discussed more in detail under emotional consequences.

### 3.3. Emotion Regulation Strategies

We defined emotion regulation strategies as actions taken by the coaches to influence their emotional experiences. These strategies can be divided into four categories: communication strategies, avoidance strategies, cognitive strategies, and bodily relaxation strategies.

#### 3.3.1. Communication Strategies

Sixteen coaches in this study reported employing communication strategies to regulate their emotions. This means that each of these football coaches communicated or expressed their emotions to their players or to others with whom they shared interpersonal relationships. Communication strategies manifest in two ways: verbal communication and expression.

Ten coaches noted that sharing individual thoughts and emotions with their players, friends, families, and referees influenced their emotions. In other words, to regulate their emotions, they discussed their emotions with other people. One coach described his use of this strategy as follows:

I certainly have very intense conversations with the team. I also discuss my feelings with my wife in the evening. I told her how much the situation annoyed me…. I believe talking is gold and silence is silver. I can develop and gain very much from the conversations.

(I8)

Ten coaches regulated their emotions by expressing them. They expressed their emotions by providing positive feedback and showing their emotion to their players and teams. Illustrating his strategies for providing a player with positive feedback and showing emotions, one coach commented:

I have shown my emotions to the players by clapping my hands and expressing positive emotions externally, of course, keeping especially the player in the limelight.

(I12)

#### 3.3.2. Avoidance Strategies

Eleven coaches used avoidance strategies. This means that the coaches did not express their emotions to their players. Instead, they repressed their emotions or kept a distance from emotionally triggering situations and emotional experiences. Avoidance strategies are characterized by distraction, use of narcotic substances, and suppression.

Instead of communicating, eight coaches preferred to distract themselves from certain situations and emotions. Distraction involves shifting the focus away from situations that trigger positive or negative emotions. For example, some coaches preferred not to enjoy positive emotions by focusing on their tasks. One coach highlighted this strategy:

There are some happy experiences, but there may be also not such happy experiences. Life goes on. Success is therefore not continuous. A new season comes and you have to pay attention to other things as you just need to do it again.

(I4)

This comment shows that coaches regulate positive emotions to effectively complete their tasks. Two coaches engaged in hobbies—listening to music, and taking part in leisure activities—to regulate their emotions. One coach described this strategy as follows:

I sit down in front of the television. For away games, I put some music on and travel home calmly. I walk the dog in the forest.

(I5)

He also used narcotic substances—smoking tobacco and drinking beer—to regulate his negative emotions:

I do not really smoke, but sometimes after a game, I smoke a cigarette. Then, I drink a few beers.

(I5)

Additionally, three coaches in this study claimed to avoid displays of emotion. One coach described suppressing his own negative emotions during games to avoid negatively influencing his players:

I’ve bitten my tongue many times in order not to transfer my emotions, my inner emotions, towards my players.

(I7)

Another coach noted that he regulated the display of positive emotions to prevent them from negatively affecting his players:

I suppressed my positive emotion a bit because it does not suit well to my players. If I show so much of my emotion, if I am too happy as a coach or say too much “I have won”, I think that is not good for my players.

(I6)

#### 3.3.3. Cognitive Strategies

Eleven coaches in the study employed cognitive strategies to regulate their emotions. Such strategies involve the use of mental skills to resolve and enhance emotional experience. These mental skills include situational analysis, reappraisal, self-talk, and fantasy.

Seven coaches reported using situational analysis. They mentioned focusing their attention on problematic events in order to identify the reasons why the problems had occurred, in order to regulate their negative emotions. Describing this strategy, one coach noted:

I search for the cause of the problem to find out how to prevent it and how it could work better.

(I8)

Another coach discussed this approach more in detail:

I’ve been thinking about this game. I ask myself about what has led to this result? In addition, I always write down what I have said to the team before the game. Then, I also check whether my team implemented my coaching. If they didn’t, I ask for the reason. This is how I analyze the game for myself.

(I11)

The second cognitive strategy coaches used was reappraisal. While in some ways similar to situational analysis as an approach to emotion regulation, reappraisal involves focusing on personal evaluation of a situation. Three coaches mentioned that when they felt a negative emotion, they attempted to find a meaningful outcome by approaching the triggering situation from a different perspective. One coach explained this approach as follows:

Yes, to learn from these errors, to profit from it in some way, I have to think about them again and say: Okay. The useful things must then be collected.

(I8)

Seven coaches employed two other cognitive strategies—self-talk and fantasy. They reported using self-talk and fantasy during the games to control their emotions to avoid negative influences on their players and teams. Four coaches applied self-talk, which consists of verbal and non-verbal behavior used to covertly express emotion. For example, the coaches engaged in several covert forms of behavior including swearing and clenching their fists to control their emotions. One coach described the non-verbal form of this strategy:

I even clenched my fist in my pants so that my feeling does not show to my players.

(I12)

Another coach described the strategy’s verbal form:

I try to hide my emotion. I have said some words to myself, I also really swore, but tried to do it at a low volume. Then, as I said, I became very relaxed again.

(I17)

The coaches also said that they wrote a story in their mind that was quite different from the reality. Describing such a situation, one coach commented:

If I had expressed my feeling, I believe the situation would have escalated and then it would probably have resulted in forfeiting the game.

(I2)

#### 3.3.4. Relaxation Strategies

The last category of emotion regulation strategies is relaxation. Relaxation refers to both physical and mental relief. Three coaches employed these strategies. To regulate negative emotions, the coaches applied several strategies including going to the sauna, controlled breathing, and rest. One coach explained his use of these strategies as follows:

With these possibilities, the disappointment is quickly processed. In any case, I go to the sauna. This is the thing that best resolves my emotions.

(I8)

However, six coaches reported difficulties with regulating negative emotions. One coach stated:

When I am aggressive, I somehow try to calm down again. But it is difficult for me to be calm in certain stressful situations. It is sometimes difficult to calm down again.

(I17)

Another coach attributed his difficulty to regulate emotion to his lack of knowledge of ways to regulate his emotions, remarking:

Emotion always depends on the situation, but dealing with emotions is always the same. So, one way or another, in a situation like that, there are not so many options.

(I5)

This quote suggests that coaches apply emotion regulation strategies based on their knowledge of and their familiarity with said strategies. In other words, football coaches can employ the strategies they know, reutilizing the approaches they typically use. The quote below reinforces the notion that coaches tend to rely on familiar strategies:

I’m not a man who outwardly expresses happiness about the development of his players. Instead, I keep happiness to myself and don’t even show emotions like sadness or joy. I silently mourn or feel happy for my players.

(I9)

### 3.4. Emotional Consequences

The coaches’ positive and negative emotions had emotional consequences, which we divided into four categories: emotional stability, confidence, social openness, and concentration.

#### 3.4.1. Emotional Stability

Fifteen coaches reported emotional stability. This term refers to the maintenance of stable or unstable emotional conditions. It includes two opposing poles: emotional stability and emotional instability.

Twelve coaches noted that positive emotions contributed to their emotional stability. They claimed that positive emotions helped them to become calm, gain satisfaction, and sleep better. The following quote captures this perspective:

I develop ease and inner satisfaction. The happiness leads to this general sense of well-being. I believe I am also more resilient. Dealing with criticism, perhaps also in other contexts, I accept everything in a more relaxed way.

(I8)

On the other hand, 10 coaches generally acknowledged that negative emotions had an immediate negative impact on their emotional stability. They noted that negative emotion made them experience stress, aggression, sleeplessness, and nervousness. The following quote illustrates a coach’s experience of sleeplessness and nervousness:

I know that the negative feelings lead to really bad sleep and problems with the nervous system. I was tense, and I could not sleep well, either.

(I6)

#### 3.4.2. Confidence

Nine coaches also indicated that emotion influenced their confidence. This term refers to the intensity of the coaches’ belief in their abilities, which consists of two opposite poles: high confidence and low confidence.

The coaches noted that their positive emotions led them to assess themselves and their coaching more positively. Describing the effect of positive emotions on his confidence, one coach stated:

I become more self-confident through success and happy feelings. So I realize that I am more self-confident and this also becomes clearer in my entire personality. It also clearly strengthens my personality. Again and again, all the successes and happiness I have strengthen me as a person.

(I4)

Correspondingly, another coach explained that negative emotions decreased his confidence by stating:

When I’m afraid, my aura doesn’t seem positive to my team and I become distant. It will be rather bad for me.

(I6)

#### 3.4.3. Social Openness

Six of the coaches involved in this study reported that their emotions promoted social openness. This term refers to the coaches’ tendency to socialize and to maintain social interactions with other people. Social openness occurs on two spectrums: social openness and social isolation.

Four coaches mentioned that positive emotions made them more open—an influential factor for the coaches in terms of building social relationships. When enjoying positive emotional conditions, these coaches tended to regard other people more favorably and to be more accepting of other people’s approaches. One coach explained the impact of positive emotions on his openness as follows:

I personally felt well. I was relaxed, open, simply very happy and friendly towards the other people because I felt so good.

(I14)

Five coaches noted that, unlike positive emotions, negative emotions had negative social impacts. For instance, while experiencing negative emotions, the coaches tended to intentionally avoid interaction with their players and others. Describing this impact on his social isolation, one coach noted:

When I was not ready for disappointment, I withdrew. I did not want to be comforted or talk to anyone, and I wanted to be alone.

(I1)

#### 3.4.4. Concentration

Two coaches mentioned that their emotions impacted their concentration. This category refers to the coaches’ ability to focus their attention on an objective. It occurs on two spectrums: high concentration and low concentration.

One coach pointed out that positive emotions increased his ability to focus on tasks. Explaining how being in a positive emotional state affected his concentration, he remarked:

I was not in a negative state…. I was clearly and calmly aware of my tasks.

(I10)

He also explained the negative impact of negative emotions on his concentration, stating that experiencing negative emotions made it harder for him to concentrate:

I cannot sincerely do my task and implement my own principles.

(I10)

One coach explained that being in a sad emotional state made it difficult for him to discern whether a situation was positive or negative. He remarked:

At the moment, the situation is difficult for me. My wife died two years ago, so at the moment, I would not be able to answer which situations are positive and which are negative for me because of my personal sad situation.

(I9)

The above quotes suggest that the coaches’ emotional state may affect their judgement during situations they encounter.

## 4. Discussion

This study analyzed specific emotional processes of coaches in football. We developed a model explaining emotions as a circular process, which describes the arousal and experience of emotions of coaches, how these emotions can be managed, and how they affect the coaches. These findings could help scholars and coaches to more generally understand the emotional processes. They can also inform how the coaches’ abilities to process emotions can be developed. This section explains how the four components of the coaches’ emotional processing identified in this study (e.g., triggers, emotional experiences, emotion regulation strategies, and emotional consequences) connect to previous research and theories of EC.

### 4.1. Triggers

Our study determined that various factors including success, social relationships, leadership, and support trigger emotions in football coaches. This finding helps to explain the coaches’ priorities in football contexts because individual perspectives determine cognitive appraisals of situations [38]. Previous studies have shown that team results, inter-team relationships (these between coaches and players, parents, and managers), and the relative supportiveness of these relationships can evoke negative emotions in coaches [39,40]. While these previous studies focused on negative emotions only, our study also took positive emotions into consideration. The interesting finding is that football coaches emphasized not only on the results of games (i.e., whether they won or lost), but also on the meaningful performance (i.e., how their team played on the field). This perspective reveals that the value of sport comprises winning and the quality of performance [41,42]. In addition, we found that football coaches also regard sportsmanship as an important aspect of football. In this view, football coaches can promote sportsmanship and counteract negative societal development (e.g., increasing selfishness). This perspective corresponds to the campaigns of both the Fédération Internationale de Football Association (FIFA) and Union of European Football Associations (UEFA), supporting fair play and respect and combating racism and discrimination. In contrast to previous studies examining the stressors of professional coaching, the support which triggers emotions in football coaches was limitedly mentioned by the coaches who participated in our study and who work in the semiprofessional and amateur areas. This can partly be explained by the coaches in our study work in the amateur area. This assumption is supported by Taylor [43], stating that the situation can be encountered differently according to the coaches’ professional or amateur level. In other words, the situations coaches encounter can vary according to their positions on the amateur–professional spectrum. Our examination of these four triggers suggests that football leagues and organizations should seek to foster a culture that emphasizes not only the results of the game, but also the quality of performance and mutually respectful relationships among the participants.

According to the perspective mentioned above, four components are the main emotional determinants of football coaches’ emotions. Awareness of the triggers of emotions helps coaches to perceive emotions more accurately. This is because each emotion is generated, depending on an individual’s appraisal of a given situation [5]. Previous studies have demonstrated the importance of emotional triggers. These who are aware of their emotional triggers can accurately perceive and effectively regulate their emotions [44]. They can also recover from having negative emotions and return to positive emotional states, whereas those who lack this awareness may remain in negative emotional states [45]. In short, a comprehensive understanding of emotional triggers enables coaches to perceive what they feel and it helps them to determine what they should focus on to regulate their emotions. EC models regard this as a significant component of EC and refer to it as the understanding of one’s own emotions [13], and as comprehension of emotions [12].

### 4.2. Emotional Experiences

In this study, coaches experienced not only positive and negative emotions, but also recognized physiological, cognitive, and behavioral changes. The coaches reported experiencing the positive and negative emotions in the ways of negative thoughts, body language, tone of voice, increased heart rate, etc. [3]. These characteristics of emotional experience have been widely acknowledged for a long time. For instance, studies have demonstrated that emotional experience occurs in discrete categories recognizably based on physiological and behavioral changes [46], and facial muscle changes [47]. Research has also shown that the function of these changes relates to the intensity of emotion [48]. Plutchik [49] has integrated these perspectives into a model, in which emotions emerge in response to emotional cues occurring at the psychological, physiological, and behavioral levels, and in which these levels are collectively coordinated. Taylor [43] brought this perspective into the domain of coaches. He explained that emotion can be expressed in three main ways, including physiological response (e.g., increased heart rates and blood pressure), cognitive response (e.g., negative thinking and crowds a person’s mind), and behavioral response (e.g., active or inactive movement). In other words, coaches should consider their own different types of emotional experience to effectively perceive their own emotions.

However, some of the football coaches who participated in our study demonstrated limited abilities to recognize and experience the emotions they experienced. This resulted from a lack of emotional competence [14], since perceive emotions (e.g., positive, and negative emotions, and verbal and non-verbal expressions) are related to emotional ability [13,44]. For example, coaches who lack the capacity to distinguish emotions and lack the ability to recognize emotions may have difficulties in differentiating between emotions and identify changes in their emotional states. Barrett et al. [44], and Mayer and Geher [50] argued that the accurate perception of emotion facilitates adaptation to situations, more effective emotion regulation, and emotional well-being. Likewise, we discovered that coaches’ emotions were connected to the emotional consequences they experienced. We will discuss this finding at greater length below in the section on Emotional Consequences. In summary, the ability to consider various emotions allows coaches to accurately identify their emotions, helps them determine which emotions they should regulate and enables them to maintain well-being. EC models identify this ability as either perception of emotion [13] or identification of one’s own emotion [12].

### 4.3. Emotion Regulation Strategies

The coaches interviewed in this study used communication, avoidance, cognitive and relaxation strategies to regulate their positive and negative emotions and to maintain effective emotional states or to alleviate negative emotional states. This finding is consistent with the emotional regulation model developed by Gross [6], which consists of an antecedent and response-focused strategy including: selection and modification of situations, attentional focus, cognitive change, and modulation of responses. Previous studies exploring the regulation of emotion in sports [3,40] have found that sports coaches either communicated or withdrew from communication with players, friends and family, and used mental (e.g., self-talk and distraction) and behavioral (e.g., relaxation and exercise) skills to cope with their emotions. However, these studies focused on negative emotions only. One study that treated both positive and negative emotions [29] only considered psychological skills (e.g., self-talk, imagination, relaxation, and goal setting). In other words, previous research has not been sufficiently comprehensive to truly help coaches in enhancing their abilities to regulate their own emotions. We found that coaches tried to reduce their positive emotions in order to effectively perform their tasks. This finding coincides with Gross’ [6] contention that the promotion or prevention of emotional consequences relies on the regulation of both negative and positive emotions.

Emotion regulation strategies undoubtedly impact coaches’ emotions. However, the coaches who participated in this study struggled to regulate their emotions. Specifically, the coaches emphasized their lack of knowledge of emotion regulation, since their applied strategies depend on the strategies they know and typically apply. This finding is consistent with the perspective that knowledge and disposition are the main prerequisites for effective emotion processes [14]. Furthermore, emotion regulation strategy can be understood as the explicit and implicit processes of emotion regulation [51]. The regular practice of explicit emotion regulation strategies can use it without awareness (implicit). Consequently, coaches need to learn and regularly use emotion regulation strategies to effectively apply to influence the course and intensity of their emotions. This can help to increase the coaching effectiveness, emotional well-being, and promote more positive emotional consequences [6,30]. EC models regard such emotion regulation strategies as advanced abilities [13]. Scholars refer to them as facilitation and regulation of emotion [15], self-control [16], and regulation and utilization of one’s own emotion [12].

### 4.4. Emotional Consequences

In our discussion of emotional experiences, we mentioned the relationship between emotional experiences and emotional consequences. The coaches in this study noted that the emotions they experienced impacted their emotional stability, confidence, social openness, and concentration. This finding coincides with the Affective Event Model [52], which shows that emotions cause emotional consequences. For example, positive and negative emotions impact attention and thinking. Such states lead to flexible and creative thinking, thereby promoting health and well-being [7]. Negative emotions tend to produce negative outcomes, such as negative thinking, reduced confidence, decreased motivation, and social isolation [3]. Conversely, positive emotions can have negative influences and negative emotions can have positive influences [9]. Previous findings, indicating positive emotions can lead to reduced effort and negative emotions can promote problem-solving efforts, support our finding [53]. In other words, individuals must consider how positive and negative emotions can be used to produce desired effects.

The knowledge on the emotions promoting optimal performance has been a subject of continual interest in sports research [9]. Specifically, Lazarus [54] pointed out that people who are aware of the consequences of their emotions are more successful in regulating their emotions. For instance, the coaches who recognize that their current emotions (e.g., positive or negative) are unhelpful can immediately take the steps to regulate their emotions. As a result, enabling coaches to understand how their emotions affect them psychologically and socially will help them determine which emotions they should increase or decrease. Mayer and Salovey [15] described this emotional ability in terms of recognizing shifts in emotional progress and understanding the implications of these shifts on performance; they treated it as a component in understanding emotion. We also discovered the effect of emotional consequences on coaches’ event evaluations. Our finding is consistent with Bower’s [55] assertion that people who maintain negative emotional states tend to focus on negative aspects in the situations they face, while people who maintain positive emotional states direct their attention to positive material. Likewise, people experiencing positive emotional states tend to evaluate their situations more optimistically and to be friendlier [56]. For instance, the coaches experiencing stable emotions like satisfaction will positively evaluate the situations they encounter, whereas, the coaches who maintain low confidence will focus on the difficulties in such situations. Our circular model can explain why it is very difficult for football coaches to come out of the vicious cycle of negative emotions. When their teams lose several matches in a row, negative emotional consequences like social isolation, low self-confidence, bad concentration, and emotional instability further reinforce negative emotional experiences and can widen the gap between the coach and the team even further. Therefore, it is increasingly difficult for a coach to leave this spiral of negative emotions and to effectively cope with the team. Impairment of the performance is the logical consequence. This cycle can also explain the hot hand phenomenon [57], where positive events increase the probability for further positive events. Positive emotions foster positive emotions and negative emotions foster negative emotions. The emotion regulation is the crucial aspect to deal with this cycle of positive and negative emotions. Effective emotion regulation strategies can help to escape from the vicious cycle of negative emotions and facilitate the riding on a wave of positive emotions (e.g., hot hand phenomenon). In short, football coaches who are aware of and care for these four components can profit by experiencing a positive circular emotional process (positive emotional state), whereas those who do not comprehend and care for them may get into a negative circular emotional process (negative emotional state).

### 4.5. Strengths and Limitations

Our study offers both strengths and limitations. Meyer and Fletcher [22] and Yoo [36] suggested that a qualitative study would be the best way to improve the understanding of sport coaches’ EC. This qualitative study focused on the football and included a comprehensive sample of 18 football coaches, whose ages and experiences varied widely. Moreover, the application of a systematic analysis based on Grounded Theory principles enabled a clear and transparent presentation of the coaches’ perspectives based on empirical data.

Although we used the perspectives of multiple practicing football coaches to develop our model of coaches’ emotional processing, it is not necessarily representative of football coaches in general. Our study focused on German football coaches and did not consider other countries or cultures; hence, our findings may have limited relevance to other cultures, since mindsets (e.g., individualism and collectivism) differ among cultures [58]. Additionally, this study did not examine the coaches’ processing of players’ emotion which is the external side of EC.

### 4.6. Implications

The benefits of EC have been widely accepted in the field of sports science. Acknowledgement of these benefits generates questions about how coaches can develop their EC and which components they need to focus on in order to increase it [30]. Our study, which applied a new approach to examine how football coaches process emotions, could contribute to these fields of inquiry. Our findings could be used to design effective EC development programs to enhance coaching abilities, the psychological well-being of coaches, and the relationships of coaches. In general, the aim of qualitative studies is to generate research questions for future studies. Therefore, future quantitative studies should evaluate the model developed in this study. In particular, studies should consider how the model’s components relate to one another and underlying mechanisms in greater detail.

## 5. Conclusions

We examined the specific components of football coaches’ emotional processing. We concluded that the four identified components (i.e., triggers, emotional experiences, emotion regulation strategies, and emotional consequences) represent a four-phase process that describes the circular emotional processing of football coaches. The proposed cyclic model of emotional processes of football coaches serves two purposes. First, the model helps to better understand the underlying processes of coaches’ emotions and their handling of emotional challenges. Based on this understanding, further research, qualitative and quantitative, can make use of our findings to analyze different aspects, such as correlation between components, and coaches’ EC in other disciplines. Second, the model provides coaches and instructors of future coaches with an understanding of crucial processes in dealing with emotions in football. By utilizing this knowledge and developing strategies to deal with these processes, coaches can be enabled to lead themselves and their teams successfully. Moreover, these components can be understood as the internal components of EC. The results could be used to help football coaches better understand and improve their EC. They also provide information about which instruments of EC are applicable for football coaches. Finally, the results could be used to bolster the education of football coaches.
